# CCL2-CCR2 axis promotes metastasis of nasopharyngeal carcinoma by activating ERK1/2-MMP2/9 pathway

**DOI:** 10.18632/oncotarget.6695

**Published:** 2015-12-20

**Authors:** Jing Yang, Xing Lv, Jinna Chen, Changqing Xie, Weixiong Xia, Chen Jiang, Tingting Zeng, Yanfang Ye, Liangru Ke, Yahui Yu, Hu Liang, Xin-Yuan Guan, Xiang Guo, Yanqun Xiang

**Affiliations:** ^1^ State Key Laboratory of Oncology in South China, Sun Yat-Sen University Cancer Center, Guangzhou, China; ^2^ Department of Nasopharyngeal Carcinoma, Sun Yat-Sen University Cancer Center, Guangzhou, China; ^3^ Department of Clinical Oncology, Hong Kong University, Hong Kong, China; ^4^ Internal Medicine Residency Program, Vidant Medical Center, East Carolina University, Greenville, NC, USA

**Keywords:** CCL2/CCR2, nasopharyngeal carcinoma, metastasis, MMP2/9, ERK1/2

## Abstract

Distant metastasis remains the major failure of nasopharyngeal carcinoma (NPC). In this study, the roles of chemokine C-C motif ligand 2 (CCL2), and its receptor chemokine C-C motif receptor type 2 (CCR2) on NPC metastasis were investigated. Serum CCL2 and CCL2/CCR2 expression level were remarkably increased in NPC patients compared to non-tumor patients by ELISA and IHC analyses. High expressions of CCL2/CCR2 were significantly associated with NPC metastasis and poor overall survival (OS). High expression of CCR2 is an independent adverse prognostic factor of OS and distant metastasis free survival (DMFS). Overexpressions of CCL2 and CCR2 were detected in high-metastatic NPC cell lines. Upregulating CCL2 and CCR2 respectively in low-metastatic NPC cell lines could promote cell migration and invasion, and exogenous CCL2 enhanced the motility in CCR2-overexpressing cells. On the other hand, downregulating CCL2 and CCR2 respectively in high-metastatic NPC cell lines by shRNA could decrease cell migration and invasion. However, exogenous CCL2 could not rescue the weaken ability of motility of CCR2-silencing cells. In nude mouse model, distant metastasis was significantly facilitated in either CCL2-overexpressing or CCR2-overexpressing groups, which was more obvious in CCR2-overexpressing group. Also, distant metastasis was considerably inhibited in either CCL2-silencing or CCR2-silencing groups. Dual overexpression of CCL2/CCR2 could activate extracellular signal-regulated kinase (ERK1/2) signaling pathway, which sequentially induced matrix metalloproteinase (MMP) 2 and 9 upregulations in the downstream. In conclusion, CCL2-CCR2 axis could promote NPC metastasis by activating ERK1/2-MMP2/9 pathway. This study helps to develop novel therapeutic targets for distant metastasis in NPC.

## INTRODUCTION

Nasopharyngeal carcinoma (NPC) is one of the most common malignancies in Southeast Asia and southern China with incidences reported as 15–50 per 100,000 [1–3]. Non-keratinizing carcinoma, the major histologic form of NPC, dominates in these high-incidence areas, with a feature of high distant metastasis rate at the time of diagnosis [4] or after initial treatment [5]. It is believed that the pathogenesis of NPC metastasis is an intricate progressive process involving the accumulation of multi-genetic alterations [6, 7], and the detailed molecular mechanisms are sparsely understood.

In the human genome, chemokine C–C motif ligand 2 (CCL2) gene is one of cytokine genes located at 17q11.2-q12. CCL2 belongs to the superfamily of secreted proteins of chemokines involved in immunoregulatory and inflammatory processes. It is a monomeric polypeptide contains a signal peptide of 23 amino acids and primarily secreted by monocytes, macrophages and dendritic cells [8]. CCL2 has been implicated in etiopathogeneses of several non-neoplastic diseases including diabetes mellitus, rheumatoid arthritis, and certain neuronal degeneration [9–11]. It was also reported as an important promoter in the development and progression of many types of tumors by stimulating cell proliferation and migration directly and/or indirectly [12]. Chemokine C-C motif receptor type 2 (CCR2) is one significant type of cell surface receptors that bind CCL2 [12]. CCR2 gene is located at 3p21.31 encoding two isoforms (A and B) of CCR2 transcript variants, and our previous work has demonstrated that only isoform B could be detected in NPC. Certain functions of CCR2 have been revealed when it binds CCL2 forming CCL2/CCR2 axis, involving formation of atherosclerotic plaques [13], insulin resistance in obesity [14], and inflammatory responses against tumors [15–17]. Recent studies focused on breast cancer, prostate cancer, pancreatic cancer, and colorectal cancer implicated CCL2/CCR2 axis participated extensively in pathogeneses of tumorigenesis and metastasis [15, 18–22]. But so far there is no research reported to uncover the underlying function of CCL2/CCR in NPC. One of our previous studies in NPC had shed a light on the relationship between the serum CCL2 (sCCL2) level of patients and their prognosis, suggesting that high sCCL2 level predicts bone invasion, post-treatment distant metastasis and poor overall survival in NPC patients [23].

Amounts of studies focused on molecular mechanisms of tumor progression have defined that the matrix metalloproteinase (MMP) family is largely involved in the metastasis process, by associating with the breakdown of extracellular matrix and tissue remodeling [24, 25]. Upregulations of MMP2, MMP7, and MMP9 have been mostly reported as enhancements to the migratory and invasive ability of cancer cells [26–33].

In this present study, the expression status CCL2/CCR2 and the clinical significance in NPCs were studied. Both overexpression and silencing of CCL2 and CCR2 were conducted respectively *in vitro* and *in vivo* to characterize the biologic effects of CCL2/CCR2 axis in NPCs. The mechanism investigation demonstrated that CCL2-CCR2 axis promotes metastasis of NPC by activating ERK1/2-MMP2/9 pathway.

## RESULTS

### CCL2 and CCR2 are frequently upregulated in NPC tissues and highly metastatic NPC cell lines, and CCL2 is remarkably increased in the sera of NPC patients

**Figure 1:**
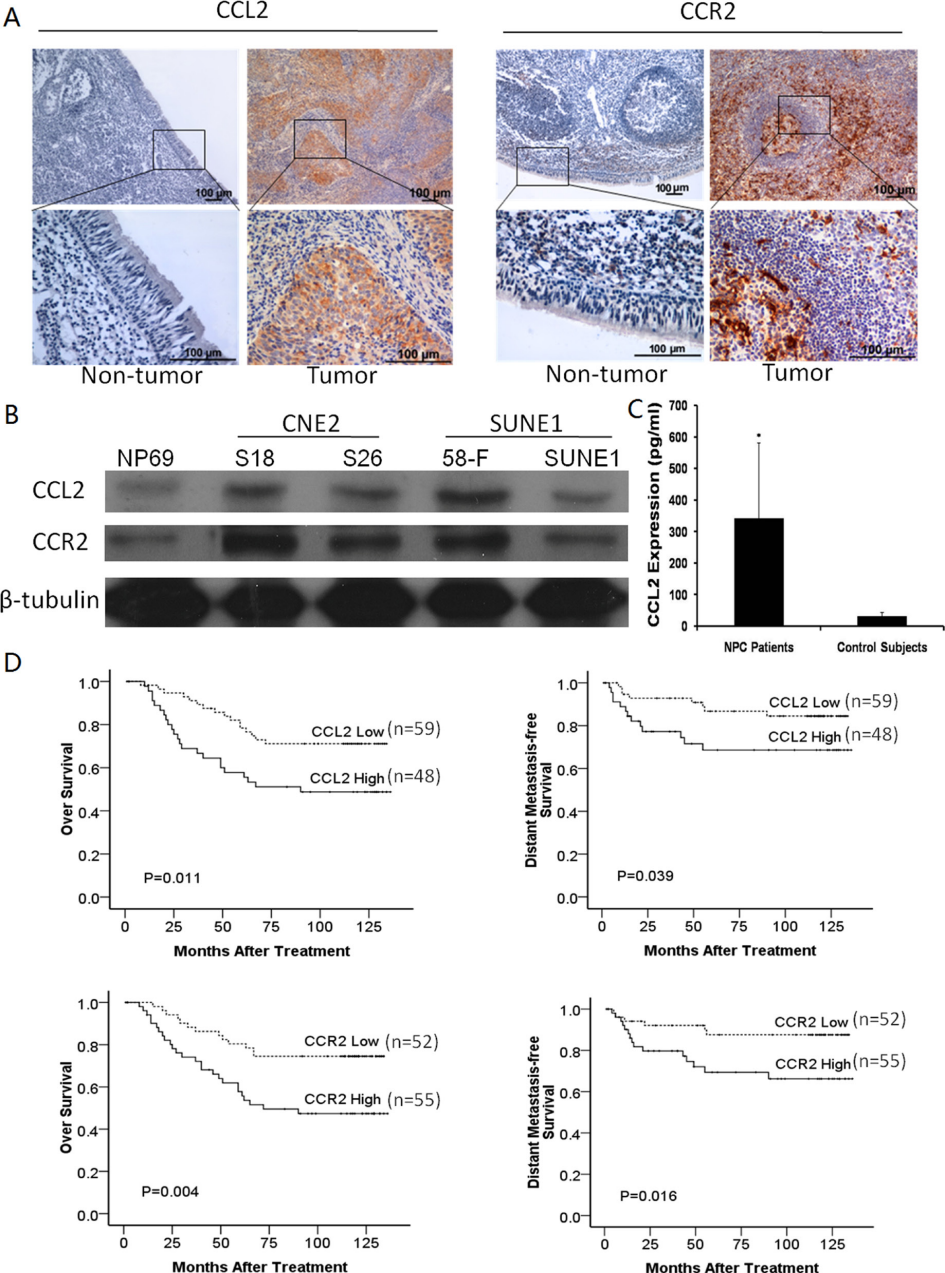
(**A**) Representative of CCL2 expression and CCR2 expression in NPC tumor tissue and non-tumor tissue detected by IHC. (**B**) Upregulations of CCL2 and CCR2 were observed in different NPC cell lines compared with non-tumor control. NP69 was set as an control. (**C**) Result from ELISA analyses of human sera, the mean serum CCL2 concentration of 50 NPC patients was significantly higher than the mean serum CCL2 concentration of 50 non-NPC patients’. *P < 0.05. (**D**) Kaplan-Meier analysis indicates both upregulation of CCL2 and upregulation of CCR2 were significantly associated with poorer overall survival and distant metastasis-free survival of NPC patients (*p* = 0.011, *p* = 0.039, *p* = 0.004, *p* = 0.016, respectively).

A cohort of tissue samples containing 50 cases of primary NPCs and 50 cases of non-tumors was studied by immunohistochemistry (IHC). Upregulation of CCL2 was detected in 48/50 (96%) in NPCs as shown in Figure 1A, compared with 0/50 in the nontumorous tissues. Similarly, upregulation of CCR2 was detected in 49/50 (98%) in NPCs, compared with 0/50 in the nontumorous tissues (Figure 1A).

Western blot analysis showed that both CCL2 and CCR2 were overexpressed in NPC cell lines compared with immortalized nasopharyngeal epithelial cell line NP69. The overexpressions of CCL2 and CCR2 were much more obvious in highly metastatic cell lines (S18 and 5-8F) compared with poorly metastatic cell lines (S26 and SUNE1), (Figure 1B).

ELISA analyses of human sera from 50 cases with NPC and their non-tumor counterparts showed that the mean serum CCL2 concentration of NPC patients (342.3 ± 238.3 pg/ml, range 106.2 pg/ml-1448.6 pg/ml) was significantly higher than the mean serum CCL2 concentration of non-tumor patients (20.0 ± 7.0 pg/ml, range 15.0 pg/ml-45.3 pg/ml), (*P* < 0.05, Figure 1C).

### Clinical significances of the high expression levels of CCL2 and CCR2 in NPC

To investigate the underlying clinical significance of CCL2/CCR2 axis, the associations of CCL2/CCR2 expression levels with clinicopathological features in 107 NPCs (informative IHC cases) were analyzed. The results found that high expression level of CCL2 was significantly associated with N stage (*P* = 0.005), and high expression level of CCR2 was significantly associated with distant metastasis (*P* = 0.032) and prognosis (*P* = 0.001, Table 1). Among them, 28.0% (*n* = 30) of 107 patients displayed high expression of CCL2/CCR2 axis (both positive of CCL2 and CCR2). Kaplan-Meier analysis indicated that higher CCL2 or CCR2 expression was significantly associated with poorer OS (log-rank test, *P* = 0.011 and *P* = 0.004, respectively) and lower DMFS (log-rank test, *P* = 0.039 and *P* = 0.016), (Figure 1D). The high expression level of CCL2/CCR2 axis was also significantly associated with distant metastasis, progression and death (*P* = 0.023, 0.036 and 0.019, respectively). Cox multivariate analysis including age, gender, WHO histological grade, T stage, N stage, clinical stage, CCL2 and CCR2 status was performed, which revealed that overexpression of CCR2 was one of the independent prognostic factors of poorer OS (*P* = 0.012) and DMFS (*P* = 0.045) of NPC patients (Table 2).

**Table 1:** Clinicopathological correlation of CCL2 expression and CCR2 expression in NPC

Clinical factor	Cases (*n* = 107)	CCL2 expression		*P* value	CCR2 expression		*P* value
		High (*n* = 48)	Low (*n* = 59)		High (*n* = 55)	Low (*n* = 52)	
Sex							
male	83 (77.6%)	40	43	0.143	42	41	0.854
female	24 (22.4%)	8	16		13	11	
Ages (years)							
< 50	67 (62.6%)	25	42	0.065	37	30	0.496.
≥ 50	40 (37.4%)	23	17		18	22	
T stage							
T1 + T2	60 (56.1%)	26	34	0.589	29	31	0.484
T3 + T4	47 (43.9%)	22	25		26	21	
N stage							
N0 + N1	78 (72.9%)	41	37	***0.005***	40	38	0.953
N2 + N3	29 (27.1%)	7	22		15	14	
Clinical stage							
I + II	45 (42.1%)	25	20	0.138	21	24	0.382
III + IVa + b	62 (57.9%)	23	39		34	28	
Local-regional relapse							
No	91 (85.0%)	41	50	0.810	43	48	0.056
Yes	16 (15.0%)	7	9		12	4	
Distant metastasis							
No	85 (79.4%)	34	51	0.062	39	46	***0.032***
Yes	22 (20.6%)	14	8		16	6	
Progression							
No	70 (65.4%)	26	44	0.080	28	42	***0.001***
Yes	37 (34.6%)	22	15		27	10	
Death							
No	67 (62.6%)	24	43	***0.016***	28	39	***0.011***
Yes	40 (37.4%)	24	16		27	13	

Statistical significance (*p* < 0.05) is shown in bold and italic.

**Table 2:** Cox proportional hazard regression analyses for 10-year OS and 10-year DMFS

Prognosis factor	Wald	Sig	HR	95.0% CI for Exp (B)	
				Lower	Upper
OS					
Age	1.601	0.206	1.629	0.765	3.471
Gender	3.375	0.066	2.466	0.941	6.461
WHO	0.398	0.528	0.673	0.197	2.302
T stage	9.149	***0.002***	3.108	1.491	6.481
N stage	11.433	***0.001***	2.452	1.458	4.123
Clinical stage	7.956	***0.005***	0.306	0.135	0.697
CCL2	0.458	0.499	1.283	***0.623***	2.643
CCR2	6.350	***0.012***	2.570	1.233	5.355
DMFS					
Age	1.780	0.182	1.998	0.723	5.519
Gender	3.090	0.079	3.870	0.856	17.496
WHO	0.600	0.438	0.443	0.056	3.475
T stage	2.976	0.084	2.382	0.889	6.387
N stage	5.734	***0.017***	2.456	1.177	5.124
Clinical stage	2.990	0.084	0.380	0.127	1.138
CCL2	0.308	0.579	1.331	0.484	3.659
CCR2	4.023	***0.045***	2.869	1.024	8.037

Abbreviations: OS, overall survival; DMFS, distant metastasis-free survival; CI = confidence interval; HR = hazard ratio.

Statistical significance (*p* < 0.05) is shown in bold and italic.

### Overexpression of either CCL2 or CCR2 promotes the migration and invasion of poorly metastatic NPC cells without influencing general cell growth, contact-independent cell growth, and anchorage-independent cell growth

**Figure 2:**
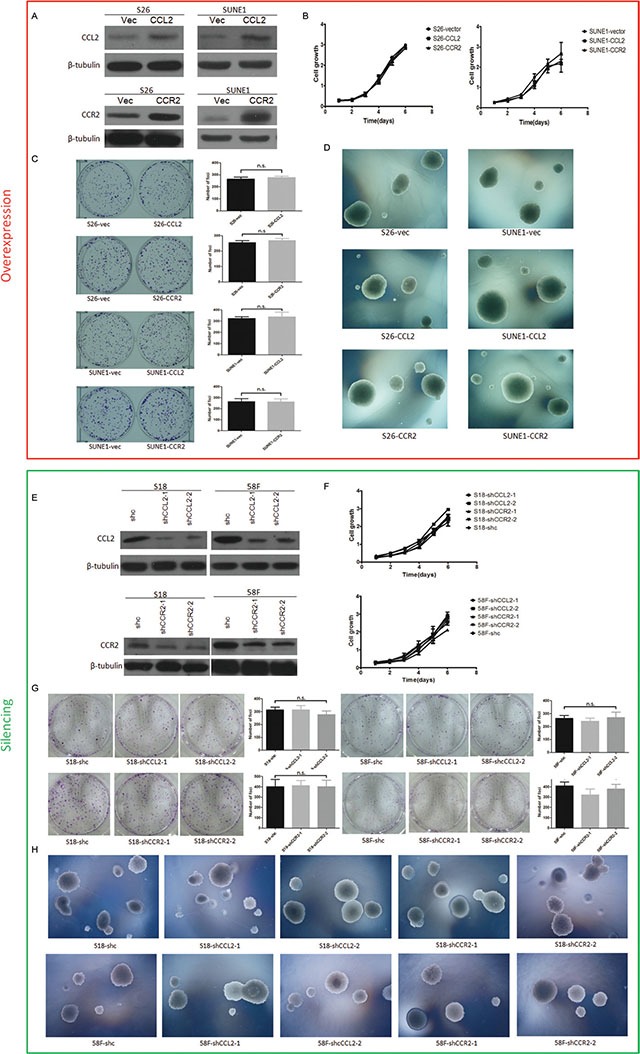
(**A**) Relatively high expressions of CCL2 and CCR2 were respectively confirmed by Western blotting in CCL2/CCR2- overexpressed S26 and SUNE1 cells compared with vector control cells. (**B**) Cell growth rates between CCL2-, CCR2- and empty vector-transfected cells were compared by XTT assay. (**C**) Representatives and summaries of foci formations in monolayer culture induced by CCL2 and CCR2 and its vector control. (**D**) Representatives of soft agar assays for colony formation induced by CCL2 and CCR2 and its vector control. (**E**) Decreased expressions of CCL2 and CCR2 were respectively confirmed by Western blotting in CCL2/CCR2- silenced S18 and 58F cells compared with scramble shRNA control cells. (**F**) Cell growth rates between CCL2-, CCR2- and scramble shRNA control cells were compared by XTT assay. (**G**) Representatives and summaries of foci formations in monolayer culture induced by CCL2 and CCR2 and its scramble shRNA control cells. (**H**) Representatives of soft agar assays for colony formation induced by CCL2 and CCR2 and its scramble shRNA control cells.

To determine the oncogenic function of CCL2 and CCR2, they were separately overexpressed in two NPC cell lines (S26 and SUNE1). Ectopic expressions of CCL2 and CCR2 were determined by Western blotting (Figure 2A).

**Figure 3:**
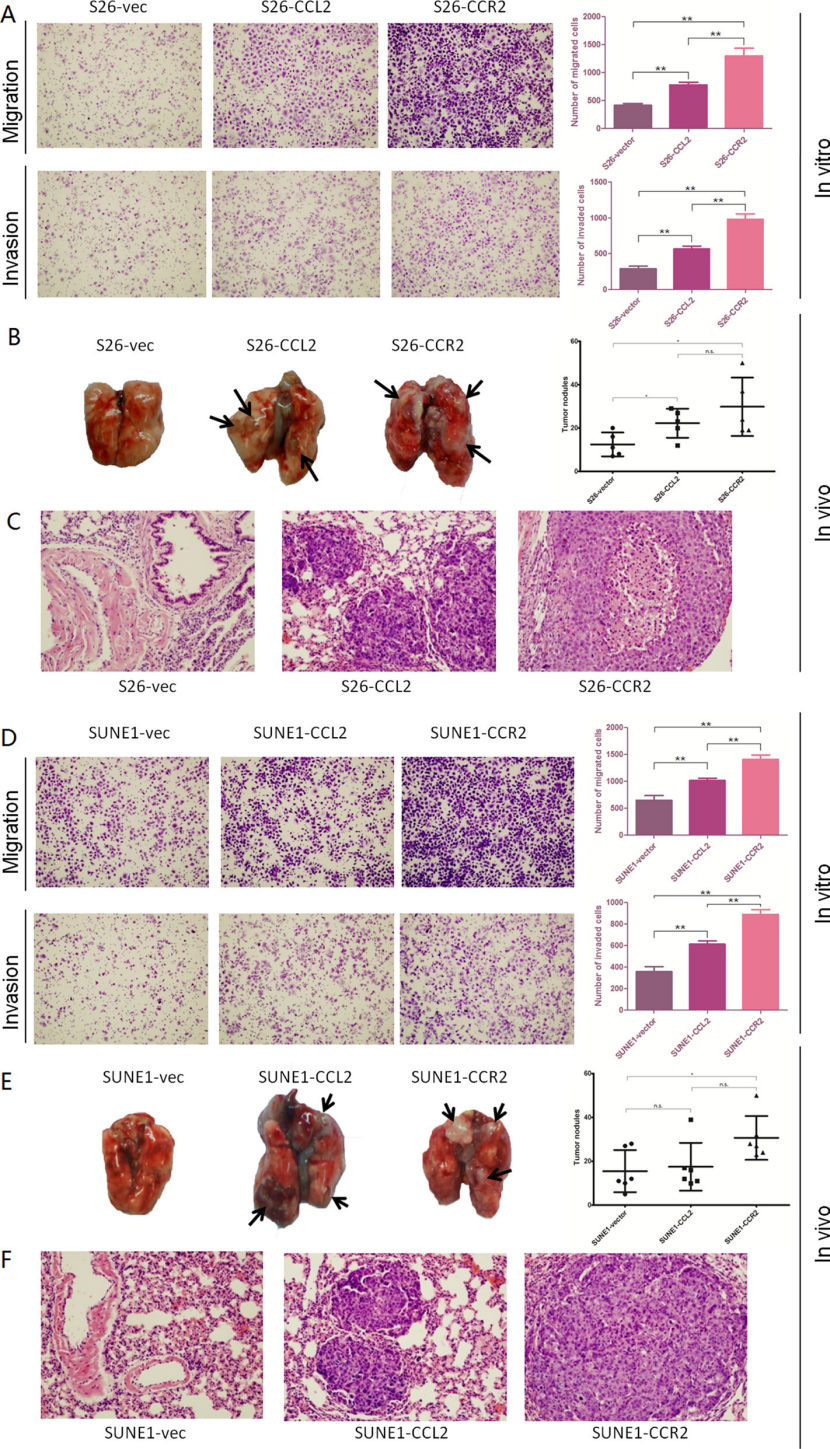
(**A**–**D**) Overexpression of CCL2 and overexpression of CCR2 respectively promotes the migration and invasion of poorly metastatic NPC cells. Representative pictures and summaries showed that both CCL2 and CCR2 could promote cell migration and cell invasion in S26 and SUNE1 cells compared with vector control cells. *P < 0.05, **P < 0.01. (**B**–**E**) Overexpression of CCL2 and overexpression of CCR2 respectively increases distant metastasis *in vivo*. Representative pictures of lungs derived from mice injected with CCL2-, CCR2- and empty vector-transfected S26 cells and SUNE1 cells. Visible tumor nodules were counted and summarized. *P < 0.05. (**C**–**F**) H&E staining was performed on pulmonary sections derived from mice. Original magnification: 20 × objective.

Functional assays including cell growth curves, foci formation and colony formation in soft agar were applied to determine the tumorigenicity of CCL2/CCR2. None of them showed CCL2 or CCR2 could promote NPC cell growth rate, tumor formation in contact-independent cell growth or anchorage-independent cell growth (Figure 2B, 2C and 2D). However, transwell of migration assays indicated either CCL2- or CCR2- overexpressed cells not only migrated more than control cells during the same time, but also invaded Matrigel more and quicker compared with their controls in invasion assays (Figure 3A and 3D).

### Overexpression of either CCL2 or CCR2 increases distant metastasis of NPC mice models

To evaluate the *in vivo* effects of CCL2 and CCR2 on tumor metastasis, same amount of S26-CCL2, S26-CCR2 or S26-vec cells were injected into nude mice (6 mice per group) intravenously through the tail vein, respectively. After 5 weeks, the mice were sacrificed and metastatic nodules at surface of lungs and livers were counted. The results showed that the number of metastatic nodules formed at the surface of the lungs of S26-CCL2 and S26-CCR2 were significantly higher than the number of S26-vec (*P* < 0.05, *P* < 0.01, respectively, independent student's *t* test. Figure 3B).

To cross-verify the role of CCL2 or CCR2 promoting distant metastasis *in vivo*, same procedures as described above were applied to mice models with SUNE1-CCL2 or SUNE1-CCR2 or SUNE1-vec cells. A similar but not identical outcome showed that, the number of metastatic nodules formed at the surface of the lungs of SUNE1-CCR2 was significantly higher than the number of SUNE1-vec (*P* < 0.05, independent student's *t* test), while the amount of SUNE1-CCL2 showed no difference from SUNE1-vec (Figure 3E). Hematoxylin and eosin (H&E) staining confirmed that the nodules on the surfaces of mice lungs were metastatic tumors (Figure 3C and 3F).

### Silence of either CCL2 or CCR2 inhibits the migration and invasion of highly metastatic NPC cells without influencing general cell growth, contact-independent cell growth and anchorage-independent cell growth

To further confirm if CCL2 or CCR2 influence cell mobility in migration and invasion without affecting tumor formation, silencing either CCL2 or CCR2 in two highly metastatic cell lines (S18 and 5-8F) was performed by RNA interference (RNAi) with two shRNAs targeting CCL2 (shRNA-CCL2-1 and shRNA-CCL2-2) or CCR2 (shRNA-CCR2-1 and shRNA-CCR2-2). A scramble shRNA was used as a control (shc). Western blotting showed that significant deduction of CCL2/CCR2 in protein level was detected (Figure 2E).

**Figure 4:**
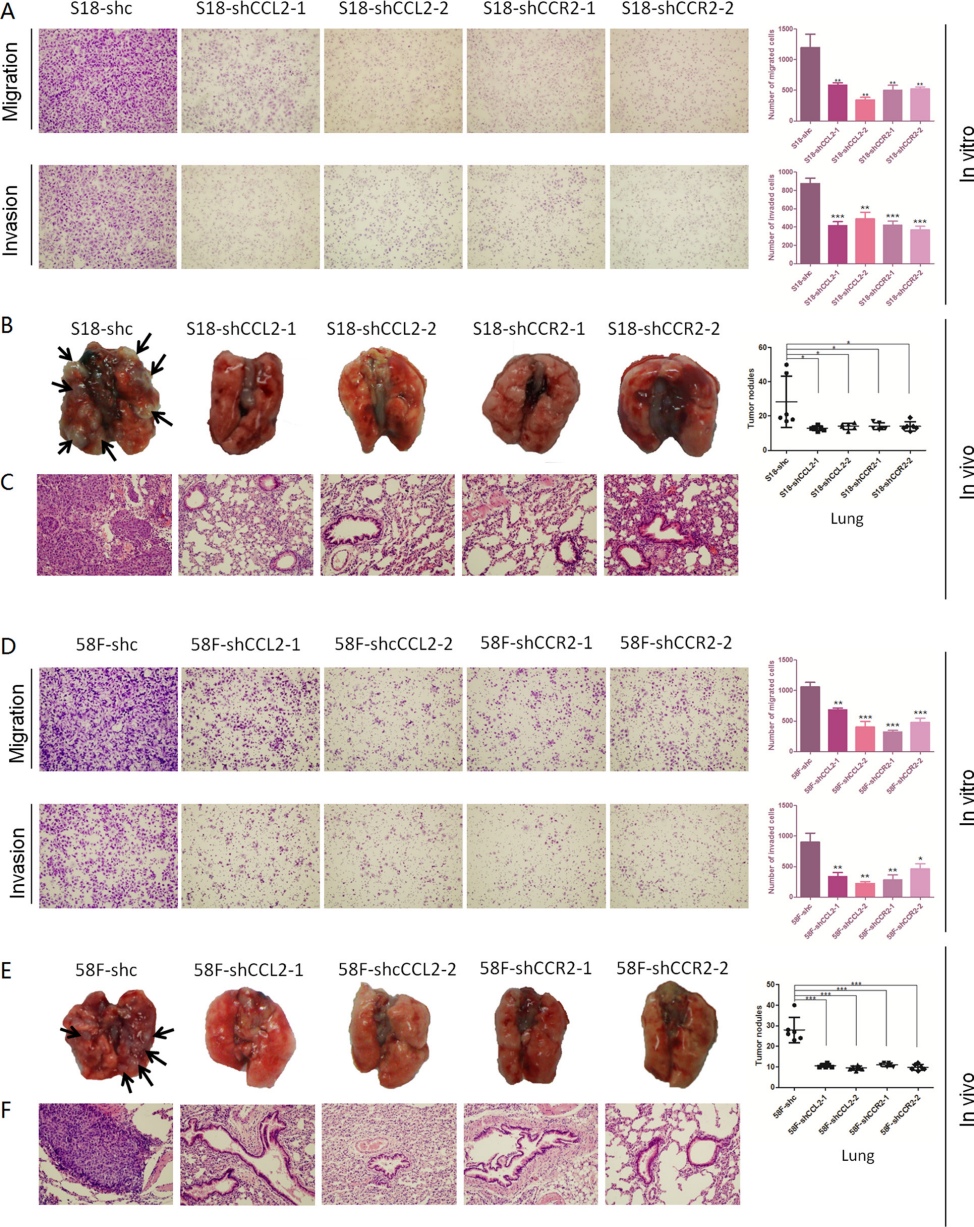
(**A**–**D**) Silencing CCL2 and silencing CCR2 respectively inhibits the migration and invasion of highly metastatic NPC cells. Representative pictures and summaries showed that silencing CCL2 and silencing CCR2 respectively could inhibit cell migration and cell invasion in S18 and 58F cells compared with scramble shRNA control cells. *P < 0.05, **P < 0.01, ***P < 0.001. (**B**–**E**) Silencing CCL2 and silencing CCR2 respectively decreases distant metastasis *in vivo*. Representative pictures of lungs derived from mice injected with shCCL2-, shCCR2- and scramble shRNA control S18 cells and 58F cells. Visible tumor nodules were counted and summarized. *P < 0.05, ***P < 0.001. (**C**–**F**) H & E staining was performed on pulmonary sections derived from mice. Original magnification: 20 × objective.

Functional assays of cell growth curves, foci formation and colony formation in soft agar revealed that NPC cell growth rate, the contact-independent cell growth, and the anchorage-independent cell growth were not significantly interfered in CCL2/CCR2 silencing cells, compared with control cells (Figure 2E, 2G and 2H). Nevertheless, in both cell lines, CCL2-silencing and CCR2-silencing performed significantly attenuated ability of migration and invasion than control cells (Figure 4A and 4D).

### Silence of either CCL2 or CCR2 decreases distant metastasis of NPC mice models

To authenticate moreover if CCL2/CCR2 silencing in highly metastatic cells could inhibit the *in vivo* metastasis, same amount of shRNA-transfected cells (S18-shRNA-CCL2-1, S18-shRNA-CCL2-2, S18-shRNA-CCR2-1 or S18-shRNA-CCR2-2 and 58F-shRNA-CCL2-1, 58F-shRNA-CCL2-2, 58F-shRNA-CCR2-1 or 58F-shRNA-CCR2-2) and their controls (shc) were injected into nude mouse (6 mice per group) intravenously through the tail vein, respectively. Organs including the lungs and livers of the mice were harvested after 5 weeks. The results showed that silencing either CCL2 or CCR2 could remarkably inhibit distant metastasis of NPC cells, mostly in lungs. Similar results were found in both cell lines (Figure 4B and 4E). H&E staining was performed to validate the nodules on the surfaces of mice lungs were metastatic tumors (Figure 4C and 4F).

### Overexpression of CCL2/CCR2 axis further enhances the migration and invasion of poorly metastatic NPC cells

**Figure 5:**
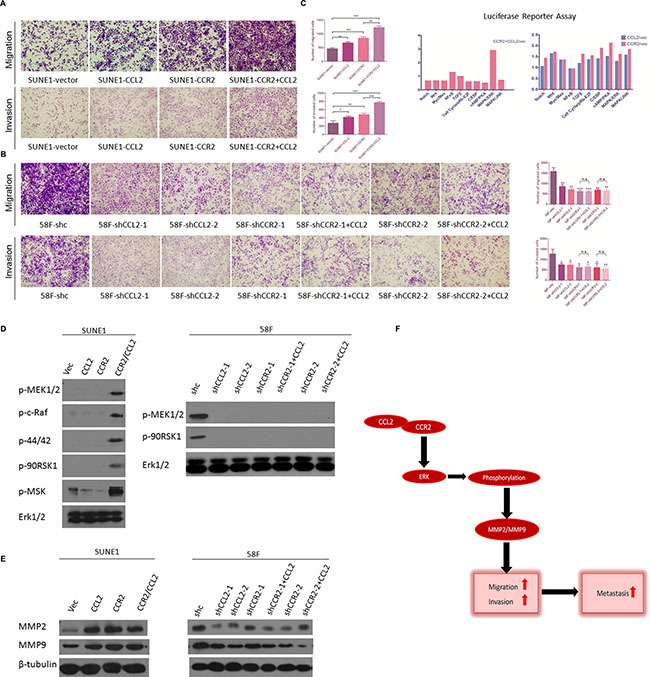
(**A**) Overexpression of CCL2/CCR2 axis further facilitates the migration and invasion of poorly metastatic NPC cells. Representative pictures and summaries showed that adding exogenous CCL2 to CCR2-overexpressed cells could intensively promote cell migration and cell invasion in SUNE1 cells compared with vector control cells and individual CCL2/CCR2-overexpressed cells. *P < 0.05, **P < 0.01, ***P < 0.001. (**B**) The suppressive motility of cell migration and invasion of highly metastatic NPC cells inhibited by silencing CCR2 were failed to be rescued by exogenous CCL2. Representative pictures and summaries showed that adding exogenous CCL2 to CCR2-silenced cells could not promote cell migration and cell invasion in 58F cells compared with scramble shRNA control cells, and individual shCCL2/shCCR2 cells. *P < 0.05, **P < 0.01, ***P < 0.001. (**C**) Luciferase reporter assay was performed separately in cells of CCL2/CCR2 overexpressed integrally (left) and respectively (right). Remarkable activation of ERK1/2 signaling pathway is observed in CCL2/CCR2 axis integrally upregulated while no transcriptional activity is distinctive when CCL2/CCR2 respectively overexpressed. (**D**) Western blotting showed that five phosphorylated key-proteins of ERK1/2 signaling pathway were increased in CCL2/CCR2 axis overexpressed SUNE1 cells compared with vector control cells and individual CCL2 or CCR2-overexpressed cells (left). Total ERK1/2 was set as an internal control. And only two of the five phosphorylated key-proteins of ERK1/2 signaling pathway were detected in 58F scramble shRNA control cells and all five were under detectable in 58F CCL2/CCR2-silenced cells (right). (**E**) MMP2 and MMP9 were compared between CCL2/CCR2 axis-, individual CCL2/CCR2- and vector-transfected cells in SUNE1 (left), or between shCCL2/CCR2- and shCCR2-cultured with exogenous CCL2, and scramble shRNA control cells (right) in 58F by Western blotting. β-Tubulin was used as a loading control. (**F**) Schematic plot of CCL2/CCR2 axis in promoting metastasis: CCL2 binds CCR2 to form activated CCL2/CCR2 axis, which phosphorylates ERK1/2 and consequently upregulates MMP2/MMP9, then increases cell migration and invasion and eventually promotes tumor metastasis.

Since either CCL2 or CCR2 could upgrade the ability of metastasis of NPC cells individually, a further investigation to overexpress the whole CCL2/CCR2 axis by adding exogenous CCL2 in CCR2-overexpression cells (SUNE1-CCR2+CCL2) was conducted. Transwell assays of migration and invasion within subgroups of SUNE1 (SUNE1-vec, SUNE1-CCL2, SUNE1-CCR2 and SUNE1-CCR2+CCL2) showed that CCR2-overexpression cells cultured with exogenous CCL2 presented the strongest ability of both migration and invasion among them, though CCL2-overexpression or CCR2-overexpression also showed enhancement of migration and invasion compared with the vector control (Figure 5A).

### The suppression of migration and invasion of highly metastatic NPC cells by silencing CCR2 fails to be rescued by exogenous CCL2

On the other hand, to explore whether CCL2 could enhance the ability of migration and invasion without CCR2 (in CCR2-silencing cells), transwell assays without Matrigel and with Matrigel were repeated in the two highly metastatic cell lines (S18 and 5-8F). Two groups cells, S18 group (S18-shc, S18-shRNA-CCL2-1, S18-shRNA-CCL2-2, S18-shRNA-CCR2-1, S18-shRNA-CCR2-1+CCL2, S18-shRNA-CCR2-2 and S18-shRNA-CCR2-2+CCL2) and 58F group (58F-shc, 58F-shRNA-CCL2-1, 58F-shRNA-CCL2-2, 58F-shRNA-CCR2-1, 58F-shRNA-CCR2-1+CCL2, 58F-shRNA-CCR2-2 and 58F-shRNA-CCR2-2+CCL2), were investigated. The results showed that CCR2-silencing cells cultured with CCL2 migrated and invaded fewer than the controls (shc), which was parallel to CCL2-silencing cells or CCR2-silencing cells. This indicated that the suppressive competence in migration and invasion of highly metastatic NPC cells with silence of CCR2 could not be rescued by exogenous CCL2 (Figure 5B).

### Overexpressing CCL2/CCR2 axis results in increased activity of ERK1/2 signaling pathway as measured by luciferase reporter assays

To further study the potential downstream effectors modulated by CCL2/CCR2 axis, luciferase experiments were carried out using the Cignal Finder 10 Pathway Reporter Arrays and Dual-Glo Luciferase Assay System. Among the ten different pathways, transcriptional activity of ERK1/2 pathway was enhanced predominantly when CCL2/CCR2 axis was overexpressed integrally, which suggested that CCL2/CCR2 axis was involved in transcriptional regulation or phosphorylation of ERK1/2 pathway in NPC cells (Figure 5C).

### CCL2/CCR2 axis stimulates cell mobility by upregulating MMP2 and MMP9 expression via phosphorylating ERK1/2 signaling pathway

Western blot analysis was used to further confirm that overexpressing CCL2/CCR2 axis could activate ERK1/2 pathway. The phosphorylated sites of ERK1/2 signaling pathway including p-44/42, p-MEK, p-c-Raf, p-90RSK and p-MSK were detected in CCL2/CCR2-overexpressed cells (SUNE1-CCR2/CCL2) compared with the control. On the contrary, there was no phosphorylation at the key sites of ERK1/2 in CCL2/CCR2-silenced cells (58F-shCCL2-1, 58F-shCCL2-2, 58F-shCCR2-1, 58F-CCR2-1+CCL2, 58F-CCR2-2 and 58F-CCR2-2+CCL2), and for the control, only p-MEK and p-90RSK could be detected in 58F-shc cells (Figure 5D).

It has been proven by numerous studies that MMPs expression could be modulated by ERK1/2 signaling pathway in various non-oncologic diseases and cancers. Among MMPs family, MMP2 and MMP9 have been confirmed playing important role in enhancement of tumor metastasis. By detecting MMP2 and MMP9 expression levels in CCL2/CCR2-overexpressed cells (SUNE1-CCL2, SUNE1-CCR2, SUNE1-CCR2/CCL2) and CCL2/CCR2-silenced cells (58F-shCCL2-1, 58F-shCCL2-2, 58F-shCCR2-1, 58F-CCR2-1+CCL2, 58F-CCR2-2, 58F-CCR2-2+CCL2), both of them were upregulated or downregulated accordingly compared with the control (Figure 5E), which was also consistent with the expression of phosphorylated sites of ERK1/2 pathway (Figure 5F).

## DISCUSSION

It has been previously studied that, in the tumor microenvironment, inflammatory cells and molecules modulate many events of cancer progress, including tumor metastasis [34–39]. CCL2 and its receptor CCR2 are the chemokines largely participating in tumor microenvironment by regulating macrophage mobilization and infiltration [40, 41], and also by recruiting inflammatory monocytes from bone marrow to peripheral sites of inflammation [15]. Plenty of researches suggest that CCL2/CCR2 is related to poor outcome and metastatic events in several cancers [15, 18, 23, 42–44].

In this study, we investigated the metastatic enhancement of CCL2/CCR2 axis in NPC both *in vitro* and *in vivo*. Our main advantages are as following: First, the ample patient resource with 10-years follow-up from NPC endemic area, which made the outcome more reliable in respect of clinical prognosis. Second, we used two pair clones with highly metastatic and poorly metastatic potential (S18 and S26, 58F and SUNE1), which are derived from single parental cell line of each other (CNE2 and SUNE1, respectively). As reported previously, tumor metastases usually arise from rare clones in the tumor [45], thus using identified clones for comparison may disclose the pivotal molecules in tumor metastasis distinctly [46]. And most of all, we gained insights into four significant aspects of how CCL2/CCR2 influence NPC metastasis in a spatial and temporal manner as follows.

First, sufficient clinical-data in human tissues and sera showed CCL2/CCR2 were dramatically upregulated in NPC patients compared to nontumor cases, and also negatively correlated to 10 yr OS and DMFS in 107 NPC cases. This suggested that CCL2/CCR2 played an important role in the distant metastasis of NPC.

Second, the overexpression of either CCL2 or CCR2 respectively in poorly metastatic NPC cell lines (S26, SUNE1) could enhance cell migration and invasion *in vitro*, and promote metastasis *in vivo*. On the other hand, silencing CCL2 or CCR2 respectively in highly metastatic NPC cell lines (S18, 58F) could inhibit cell migration and invasion *in vitro*, and attenuate metastasis *in vivo*. Interestingly, whether upregulating or downregulating CCL2/CCR2, tumorigenesis was not be influenced in two independent cell-line pairs, arguing against the possibility that the observed increment or reduction in metastasis are the result of tumor growth rate. Based on these findings, we raise a perception that by different mechanisms tumor growth and metastasis of NPC could be modulated. CCL2/CCR2 might probably act upon various steps of metastasis rather than tumorigenesis. In line with our results, previous work from Sawanobori et al. also indicated that CCR2 deficiency did not affect primary tumor growth [42, 47]. Recent observations that CCL2 overexpression in tumor cells increased metastasis [42, 48] are in accordance with our findings.

Third, NPC cell mobility could be tremendously facilitated in functional studies when the expression of CCL2 and CCR2 was overexpressed as an integrated axis by adding exogenous CCL2 in CCR2-overexpressed cells, and a validation *in vivo* is expected in our further study. On the other hand, the inner interaction within CCL2/CCR2 axis was also unveiled to draw a map that, CCR2 would probably be the onset-limiting part in the axis, for exogenous CCL2 failed to rescue the inhibited cell migration and invasion when CCR2 was insufficient. This finding coincides with a significant outcome of a profound research in colon carcinoma [42].

Last but not least, upregulation of MMP2 and MMP9 through ERK1/2 signaling pathway was identified as the metastasis-promoting mechanism of CCL2/CCR2 axis in NPC. It is well-known that the MAPK pathways (i.e. ERK1/2, JNK, and p38) participate in numerous signaling cascades that play regulatory roles in cell growth, apoptosis, differentiation, and metastasis [49]. Recently, there are several researches in Hela cells, breast cancer, colon carcinoma and melanoma showing CCL2/CCR2 axis is related to the activation of MAPK pathways in ERK1/2, p38, and JNK [42, 50–52]. It has also been reported that ERK1/2 could regulate expression levels of MMP2 and MMP9 in cancer cells, which can degrade extracellular matrix to promote metastasis in tumor [53–56]. Similarly, we identified CCL2/CCR2 axis as a tumor promoter in NPC metastasis through upregulating MMP2/9 via ERK1/2 pathways. Besides, in other studies published recently, CCL2/CCR2 axis also has been found to interact with STAT, JAK, and Smad signaling [42, 51, 57, 58], implying it might participate in extensive biological activities. Interestingly, an incomprehensive finding of the inconsistency of metastasis mechanisms in CCL2/CCR2 axis activated integrally and individually was observed. Cignal Finder 10 Pathway Reporter Arrays of CCL2- or CCR2-overexpressed cells showed no obvious change in transcriptional activity of the ten pathways, with only minor increase in Wnt, cAMP/PKA and MAPK/JNK. Intriguingly, ERK1/2 signaling pathway was distinguished with high transcriptional activity when CCL2/CCR2 axis was overexpressed integrally by adding exogenous CCL2 in CCR2-overexpressed cells. The immunoblotting analysis of ERK1/2 pathway also demonstrated its activation.

Lately the antagonists to CCL2 and CCR2 are available and some of them are being used in clinical trials, which can be expected in the treatment of distant metastasis of NPC. Recently, however, one remarkable study published on *Nature* alerted that, cessation of CCL2 inhibition would accelerate breast cancer metastasis in mice [59]. Though this phenomenon is not widely observed in other cancers yet, attention should be aroused when suppressing CCL2/CCR axis is applied for tumor treatment. Taking this distinctive occurrence and our findings together, we modestly suggest that, under the circumstance of consecutive and steady administration, inactivating CCL2/CCR2 axis, especially CCR2, would be a promising treatment of NPC.

The limits of our study are also concerned. We failed to establish a stable cell line to overexpress CCL2 and CCR2 simultaneously, instead, we added exogenous CCL2 in the culture medium of CCR2-overexpressed cells to imitate the overexpression of CCL2/CCR2 axis *in vitro*. Also, we did not carry out animal models to confirm the promoted mobility or metastasis of cells overexpressing CCL2/CCR2 axis due to limited fund. On the other side, we did not investigate the function of silencing CCL2/CCR2 axis integrally *in vitro* or *in vivo*.

In conclusion, CCL2/CCR2 axis plays an important role in the promotion of NPC metastasis by upregulating MMP2 and MMP9 via activating ERK1/2 pathway, which may lead to the identification of new therapeutic targets for distant metastasis of NPC.

## MATERIALS AND METHODS

### NPC samples and cell lines

A number of 50 primary NPC tumor samples and another 50 nontumorous samples were immediately collected from total 100 NPC patients who underwent nasopharyngeal biopsy in electric nasopharyngoscopy at Sun Yat-Sen University Cancer Center (SYSUCC), Guangzhou, China, before treatment. All samples used in this study were approved by the Committees for Ethical Review of Research at Sun Yat-Sen University.

Four human NPC cell lines, S18 and S26 (highly metastatic clone and poorly metastatic clone of CNE-2), SUNE-1 and its highly metastatic clone 58F, were kindly provided by Professor Qian Chaonan (Department of Nasopharyngeal carcinoma, SYSUCC, Guangzhou, China). All these cell lines were cultured in less than 20 passages, and maintained in DMEM (Dulbecco's modified Eagle's medium) supplemented with 10% FBS at 37°C. Regular morphologic observation and test for absence of mycoplasma contamination (MycoAlert, Lonza) were authenticated in all cell lines used in the present study.

### Human tissues, immunohistochemical staining and histologic evaluation

A total number of 107 formalin-fixed and paraffin-embedded NPC specimens were obtained from pathologically diagnosed patients at SYSUCC between August of 1999 and March of 2000. This cohort consisted of 83 male and 24 female patients, giving a male: female ratio of 3.46:1, and the median patient age was 47 years (range 18–91 years). Proportions of patients with late T stage (T3-4) and late N stage (N2-3) were 43.9% and 27.1%, respectively (Table 1). All patients received radiotherapy with doses of 60 to 80 Gy to nasopharynx and 50 to 80 Gy to the neck.

In immunohistochemical (IHC) analysis of CCL2 and CCR2, the paraffin-embedded slices were deparaffinized, rehydrated, and blocked by 5% bovine serum albumin (BSA) at room temperature for 20 minutes, then incubated with rabbit polyclonal antibody against CCL2 (bs-1955R, Bioss) /CCR2 (bs-0562R, Bioss) at a dilution of 1:100 at 4°C overnight, and subsequently incubated with horseradish peroxidase (HRP) anti-rabbit/mouse immunoglobulin at a concentration of 1:100 for 30 minutes at 37°C, then detected the primary antibodies followed by 3, 3-diaminobenzidine substrate visualization and counterstaining with hematoxylin (GTVision III Detection System/Mo & Rb). The IHC staining index was calculated independently by two pathologists, adding the scores for the intensity of CCL2-positive staining or CCR2-positive staining (negative, 0; weak, 1; moderate, 2; or strong, 3) and the percentage of CCL2-positive cells or CCR2-positive cells (< 25%, 1; 25%–50%, 2; > 50%–75%, 3; > 75%, 4 scores). The final score was the average value from the two referees.

Lungs and livers of nude mice with distant metastasis were formalin-fixed and paraffin-embedded and sectioned at 5 mm throughout the organs and one section in every 20 sequential sections was selected for hematoxylin and eosin staining.

### Human sera and enzyme-linked immunosorbent assay (ELISA)

From Nov 2009 to Dec 2009, 100 serum samples including 50 non-tumor patients (normal control) and 50 histologically verified NPC patients were collected before treatment in Department of NPC at Sun Yat-Sen University Cancer Center.

The concentration of serum CCL2 (sCCL2) was measured with commercially available human CCL2 quantitative ELISA kit (R&D Systems, Minneapolis, MN, USA) according to the instructions provided by the manufacturer. After the reaction, a value at wavelength of 450 nm was measured with enzyme-linked spectrophotometer, and the concentration of sCCL2 was calculated from the standard curve. All analyses were made in duplicate and the mean value was used for statistical analysis.

### Plasmid constructs and transfection

Either full-length of CCL2 cDNA or CCR2 cDNA were amplified by polymerase chain reaction (PCR) and cloned into plenti6 expression vector (Invitrogen). Lipofectamine 2000 (Invitrogen) was used to stably transfect CCL2 and CCR2 into S26 and SUNE-1 cells, respectively. Blank vector-transfected cells were used as controls.

### Establishment of CCL2 knockdown cells and CCR2 knockdown cells

Short hairpin RNAs (shRNA) in lentivirus against CCL2 (shRNA-CCL2-1, shRNA-CCL2-2) and short hairpin RNAs (shRNA) in lentivirus against CCR2 (shRNA-CCR2-1, shRNA-CCR2-2) were purchased from GenePharma Co., Ltd (Shanghai, China) and stably transfected into S18 and 5-8F cells, respectively. Scrambled shRNA-transfected cells were used as negative controls.

### Cell growth assay, foci formation assay, and soft agar assay for colony formation

For cell growth assay, cells were seeded in 96-well plate at a density of 1 × 10^3^ per well and cell growth rate was assessed by Cell Counting Kit-8 (Dojindo). Cellular growth curves were plotted by using the cellular viability values.

For foci formation assay, 1 × 10^3^ cells per well were seeded dispersedly in 6-well plate. After one-week culture, cell colonies were counted by crystal violet staining. The results are expressed as mean ± SD of three independent experiments.

For soft agar for colony formation, 0.5 ml DMEM supplemented with 10% FBS containing 1 × 10^3^ cells per well were mixed with 1.5 ml 0.6% soft agar-DMEM (10% FBS), then seeded on pre-established base agar [0.6% soft agar-DMEM (10% FBS)] in 6-well plate. Incubate assay at 37°C for three weeks then counted sphere-colonies by using a dissecting microscope. The results are expressed as mean ± SD of three independent experiments.

### *In vitro* migration and invasion assays

Migration assays were conducted with Biocoat without Matrigel (Corning. Life sciences) and invasion assays were performed with Biocoat with Matrigel (Corning. Life sciences) following the manufacturer's instructions. The reaped Biocoats were then stained with crystal violet and invaded cells were counted under a microscope. Both experiments were repeated independently in three times.

### *In vivo* distant metastasis assays

Male nude mice between 4 and 5 weeks of age were obtained from Guangdong Medical Laboratory Animal Center (Guangzhou, China). All the animal studies were conducted in accordance with the principles and procedures outlined in the guidelines of Institutional Animal Care and Use Committee at SYSUCC.

CCL2 overexpressing cells or CCR2 overexpressing cells (1 × 10^7^ for S26-CCL2; 1 × 10^7^ for S26-CCR2; 1 × 10^7^ for SUNE1-CCL2; 1 × 10^7^ for SUNE1-CCR2), CCL2 silencing cells or CCR2 silencing cells (2 × 10^6^ for S18-shCCL2-1; 2 × 10^6^ for S18-shCCL2-2; 2 × 10^6^ for S18-shCCR2-1; 2 × 10^6^ for S18-shCCR2-2; 2 × 10^6^ for 58F-shCCL2-1; 2 × 10^6^ for 58F-shCCL2-2; 2 × 10^6^ for 58F-shCCR2-1; 2 × 10^6^ for 58F-shCCR2-2), or control cells (1 × 10^7^ for S26-vec, 1 × 10^7^ for SUNE1-vec, 2 × 10^6^ for S18-shc, 2 × 10^6^ for 58F-shc) were subcutaneous injected intravenously through the tail vein in the mice, respectively. Distant metastases in lungs and/or livers were checked and counted after 5 weeks when mice were sacrificed. Lungs and livers were excised and embedded in paraffin for further study.

### Immunoblotting

Western blot analyses were performed with the standard protocol.

The primary antibodies, including rabbit anti-human CCL2 polyclonal antibody (#2027 Cell Signaling Technology), rabbit anti-human CCR2 monoclonal antibody (#12199 Cell Signaling Technology), phospho-Erk1-2 Pathway Kit (#9911 Cell Signaling Technology), mouse anti-human MMP2 polyclonal antibody (#4022 Cell Signaling Technology), rat anti-human MMP9 polyclonal antibody (#3852 Cell Signaling Technology), and β-tubulin monoclonal antibody (#5346 Cell Signaling Technology) were used at a dilution of 1:1,000.

### Luciferase reporter assay

Two independent assays were carried out.

SUNE1-CCL2, SUNE1-CCR2, and SUNE1-vector cells for the first assay and, SUNE1-CCR2 cells pretreated with exogenous CCL2 and SUNE1-vector cells for the second assay, were seeded in Cignal Finder 10-Pathway Reporter Array plates (QIAGEN, Dusseldorf, GER) when the cell density and cell viability met the transfection condition. Protocol of *Luciferase Cignal Finder Reporter Array Plate Format Handbook* was followed for developing the assays. Dual-Glo Luciferase Assay System (Promega, Madison, WI) was used to measured dual luciferase signals after reverse transfection.

### Statistics

SPSS statistics 19.0 was used for data analysis. Single comparisons were performed by Student's *t* test, Mann–Whitney test, or *χ* ^2^ test (2-tailed; *P* < 0.05 was considered significant). Clinical correlation study was analyzed by Pearson *χ* ^2^ test. Kaplan-Meier plots and log-rank tests were used for survival analysis. Cox's regression model was used for multivariate analysis. The median of the IHC score value was used as the cut-off point to divide the patients into high- and low-CCL2/CCR2 expression groups. Differences were considered significant when *P* < 0.05.
